# Structural Diversity in Bacterial Ribosomes: Mycobacterial 70S Ribosome Structure Reveals Novel Features

**DOI:** 10.1371/journal.pone.0031742

**Published:** 2012-02-24

**Authors:** Manidip Shasmal, Jayati Sengupta

**Affiliations:** Structural Biology and Bio-Informatics Division, Indian Institute of Chemical Biology (Council of Scientific and Industrial Research), Jadavpur, Kolkata, West Bengal, India; University of South Florida, United States of America

## Abstract

Here we present analysis of a 3D cryo-EM map of the 70S ribosome from *Mycobacterium smegmatis*, a saprophytic cousin of the etiological agent of tuberculosis in humans, *Mycobacterium tuberculosis*. In comparison with the 3D structures of other prokaryotic ribosomes, the density map of the *M. smegmatis* 70S ribosome reveals unique structural features and their relative orientations in the ribosome. Dramatic changes in the periphery due to additional rRNA segments and extra domains of some of the peripheral ribosomal proteins like S3, S5, S16, L17, L25, are evident. One of the most notable features appears in the large subunit near L1 stalk as a long helical structure next to helix 54 of the 23S rRNA. The sharp upper end of this structure is located in the vicinity of the mRNA exit channel. Although the *M. smegmatis* 70S ribosome possesses conserved core structure of bacterial ribosome, the new structural features, unveiled in this study, demonstrates diversity in the 3D architecture of bacterial ribosomes. We postulate that the prominent helical structure related to the 23S rRNA actively participates in the mechanisms of translation in mycobacteria.

## Introduction

A wealth of structural and functional studies over the last decade has led to a deeper understanding of the overall mechanism by which translation of mRNA code into peptide proceeds [1]. Nevertheless our understanding of mechanisms underlying different steps of translation is still not fully clear, reflecting the complex molecular architecture of the ribosome itself. Furthermore, while enormous amount of biochemical and genetic studies addressing the mechanism of translation has been carried out in the model organism *E. coli*, only a limited number of studies have been performed on the ribosomes of other organisms [2]–[6]. Detailed structural studies on prokaryotic ribosome are also mostly restricted for *E. coli*,and *T. thermophilus* ribosomes so far [7], [8]. Although principles of translation mechanism deduced from ribosomal structures of different organisms is expected to be the same, but it is not clear to which extent the details of translation mechanisms postulated based on studies of these organisms can be extrapolated to all other organisms.

Progress toward an understanding of the mechanism of translation in bacteria is of great importance in fighting devastating pathogenic diseases, as it has the potential to furnish clues for designing antibiotics against drug-resistant pathogenic species [9], [10]. Mycobacterium is well-known for its antibiotic resistance property. However, Mycobacterial tolerance to antibiotics has been studied mostly at cellular level since this property has been traditionally attributed to its impermeable cell wall [11]–[13]. Interestingly, many of these antibiotics (e.g. streptomycin, tetracycline, erythromycin) target translation. A detailed structural investigation on the mycobacterium ribosome may unveil new targets with drug discovery potential. In addition, structural studies on mycobacterium ribosome may allow us to piece together mechanistic models that interpret biochemical data collected on mycobacteria in structural terms.

A previous study demonstrated that both the size and the charge of the mycobacterial ribosomal proteins are considerably different from those of *E. coli* [5]. The study suggested that the divergent properties of the mycobacterial ribosomes may be related to some exceptional properties of mycobacteria, *e.g.* their slow growth. Comparison of the rRNA secondary structures with that of the *E. coli* also exhibits some distinctive features in mycobacterial rRNA.

Here, we present a cryo-EM reconstruction of a 70S ribosome from *M. smegmatis* containing a tRNA at the P-site. In comparison with the ribosomal 3D structures from other bacterial species, the density map of mycobacterial 70S ribosome shows the presence of unusual structural components. The most prominent one is a long helical structure in the large subunit near the L1-stalk related to an extra helix in the 23S rRNA secondary structure. We propose that this unusual ribosomal component is actively involved in regulating various steps of translation process in mycobacteria.

## Results and Discussion

### Overall architecture of the 70S ribosome

Cryo-EM and the single-particle reconstruction methods were employed to generate a three-dimensional (3D) map of the 70S ribosome from purified *M. smegmatis* ribosomal particles. The resolution of the map (Figure S1) was determined to be 12 Å (FSC = 0.5 criterion). We will be referring to this map in the following as the ‘*Msm*70S’. We compared the resulting density map with a previous 12.8 Å cryo-EM map of the *E. coli* 70S ribosome (here referred to as ‘*Eco*70S’) with tRNAs at the P and E sites [14]. Additionally, in order to facilitate analysis of the intermolecular contacts on the molecular level, we made reference to an x-ray structure of *E. coli* 70S ribosome [15].

**Figure 1:**
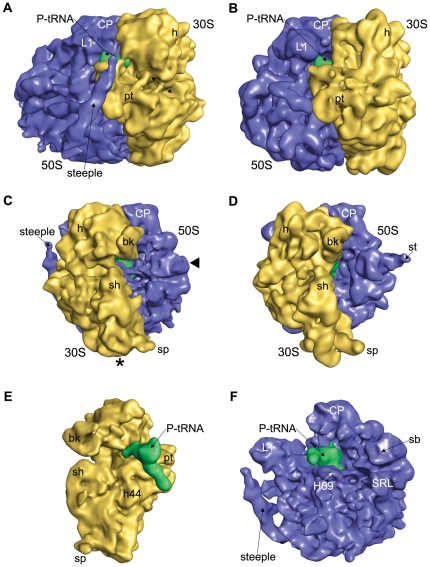
Comparison of the 70S Ribosome from *M. smegmatis* with the *E. coli* 70S ribosome. The cryo-EM map of the *Msm*70S (A, C) is shown together with the cryo-EM map of the *Eco*70S (B, D; EMD-1395). The ribosomes are shown from the L1 side (A, and B) and the L7/L12 side (C,and D). Missing extended L7/L12 Stalk is marked in *Msm*70S with solid triangle (◂). Asterisk (*) marks the missing density in the bottom part of the 30S subunit of *Msm*70S due to shorter h10 and h17. The location of the S1 and S2 proteins where corresponding densities are largely absent in Msm70S is marked with arrow. The computationally separated small subunit (E) and large subunit (F) of the *Msm*70S are shown with the P site-bound tRNA (green) from the interface sides. The small subunits are shown in yellow, the large subunits blue in all the panels. Landmarks for the 30S subunit: bk, beak; h, head; pt, platform; sh, shoulder; sp, spur; h44, helix 44 of 16S rRNA. Landmarks for the 50S subunit: CP, central protuberance; L1, L1 protein; st, L7/L12 stalk; sb, L7/L12 stalk base; SRL, sarcin ricin loop; H69, helix 69 of 23S rRNA.

The overall structure of the *Msm7*0S exhibits well defined small and large subunits (Figure 1A, C), in keeping with the feature identified in the *Eco*70S (Figure 1B, D). Morphological comparison of the *Msm*70S with the *Eco*70S demonstrates that landmark characteristics of the 30S subunit, such as head, body, and platform, are well defined in the *Msm*70S, as are the central protuberance (CP), L1 stalk and L7/L12 stalk base of the 50S subunit.

**Figure 2:**
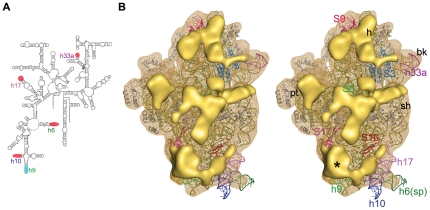
Structural analysis of the *Msm*30S. (A) Secondary structure diagram of the *M. tuberculocis* 16S rRNA. The helices which are different in mycobacterial 16S rRNA as compared to the *E. coli* 16S rRNA are marked (shorter, red; longer, cyan). (B) Stereo view of the solvent side of *Msm*30S (yellow wire mesh) with the docked crystal structure of *E. coli* 30S subunit (16S rRNA in olive, proteins in grey colour) (pdb code: 2I2U). Major extra density clusters (solid yellow) are shown. Proteins with additional segments are coloured and designated with their names. Density cluster marked with asterisk (*) represents the density corresponding to extra components of h9 and proteins S16, S17. Landmarks are as in Figure 1.

**Figure 3:**
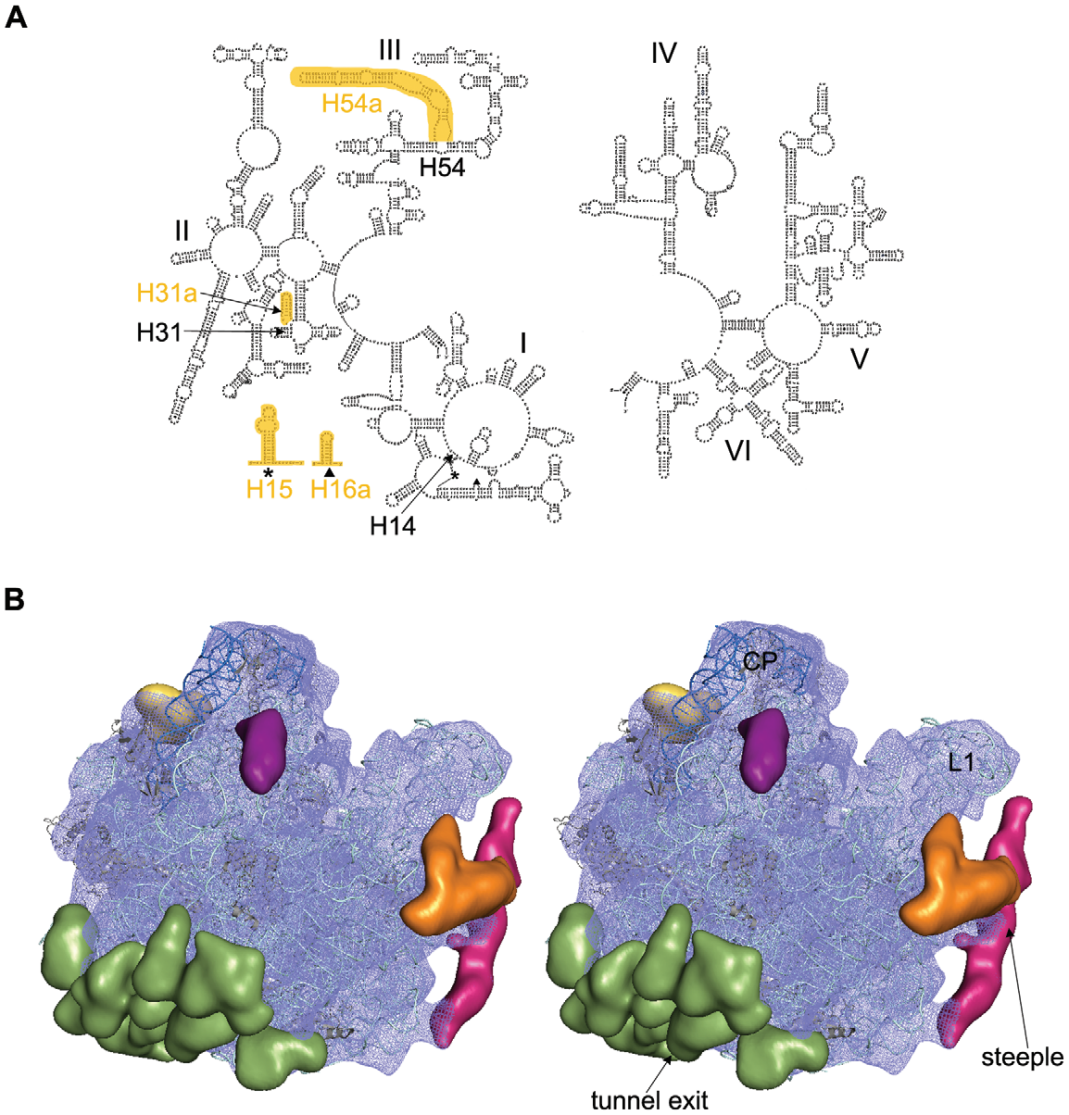
Structural analysis of the *Msm*50S. (A) Secondary structure diagram of the *M. laprae* 23S rRNA (left: 5′ end; right: 3′ end). Locations of extra rRNA helices in mycobacterium are highlighted (orange) and marked in the 5′ 23S rRNA structure (domains I, II, III). (B) Stereo view of the solvent side of *Msm*50S (blue wire mesh) with the coordinates *of E. coli* 50S subunit (23S rRNA pale cyan, 5S rRNA deep blue, proteins grey) docked inside. The atomic structure is adopted from the crystal structure of *E. coli* 70S ribosome (Protein Data Bank ID code 2I2V). Major additional density clusters are highlighted in different colors; steeple, deep pink; H15/H16a, orange; H31a, purple; additional density of L25, yellow; additional densities of proteins around the tunnel exit, green. Landmarks are as in Figure 1.

In terms of the ribosomal proteins, sequence search in ExPASy Proteomics Server (http://www.expasy.org/sprot/) indicates that almost all *E. coli* ribosomal proteins have counterparts in *mycobacteria* (except for S21), with sequence identities varying between 30% to 70%. However, some of the *M. smegmatis* small and large ribosomal proteins have longer N- or C-terminal extensions (see Table 1). Densities attributable to most of the proteins are identifiable in both the small and large subunits of *Msm*70S as reflected by the docking of the crystal structures of the *E. coli* ribosomal subunits into the density maps of the *Msm*70S subunits (Figure 2 and 3). However, either full or partial densities corresponding to some proteins are absent. The most noticeable one is the extended L7/L12 stalk in the *Eco*70S (Figure 1D) which is not visible in the *Msm*70S map (Figure 1C). Densities corresponding to S1 and S2 proteins are also largely missing from the *Msm*70S map (marked with arrow in Figure 1A). The absence of these proteins from the *Msm7*0S ribosome map might be a reflection of more dynamic nature of these proteins in mycobacteria. It may not be ruled out, however, that the purification procedure led to the removal of these proteins (known to be loosely bound to the ribosome).

**Table 1:** List of *M. smegmatis* ribosomal proteins that are bigger than their *E. coli* counter parts (proteins more than 12 amino acids (aa) longer are mentioned here).

Subunit	Proteins	Length in *E. coli*	Length in *M. smegmatis*	Extended region
	S2	241	277	**36** aa, at C-terminus
	S3	233	275	**42** aa, at C-terminus
***30S***	S5	167	214	**47** aa, in both termini
	S9	130	150	**20** aa, at N-terminus
	S16	82	156	**74** aa, at C-terminus
	S17	84	98	**14** aa, at N-terminus
	L4	201	215	**14** aa, in both termini
	L17	127	199	**72** aa, at C-terminus
***50S***	L22	110	153	**43** aa, in both termini
	L25	94	215	**121** aa, at C-terminus
	L29	63	77	**14** aa, in both termini

The cryo-EM map of the *Msm*70S clearly displays an L-shaped density in the intersubunit space (Figure 1) showing that *Msm7*0S is purified with the occupied tRNA. Juxtaposition of *Msm*70S map with the *Eco*70S coordinates containing P- and E-site tRNAs [15], identified that this density corresponds to the P-site tRNA in its entirety (Figure 1E,F). The fact that the tRNA is visible with high occupancy gives us confidence that the map represents an active ribosome.

The core of the *Msm*70S shows overall similarity with the core of the *Eco*70S while containing additional mass in the periphery. Density in the *Msm*70S map that is not accounted for by the crystal structures of *E. coli* 70S ribosome predicts largely the locations of the extra rRNA helices or of additional domains in the homologous proteins. To facilitate the docking of the crystal structure, the *Msm*70S map was computationally separated into the core and the additional density maps. The characteristic features of the subunits of *Msm*70S are elaborated below.

### Pronounced differences observed in the 30S subunit

Overall, the 30S subunit of *Msm*70S (*Msm*30S) appears shorter than its *E. coli* counterpart (Figure 1). It is observed that a piece of density is missing from the bottom part of the 30S subunit of *Msm*70S (marked with asterisk in Figure 1C) when compared to the same of *Eco*70S (Figure 1D). In the 30S subunit of *Eco*70S, h17 (for brevity, rRNA helices are denoted as ‘h’ for the 16S rRNA, or ‘H’ for the 23S rRNA) runs parallel to the long axis of the subunit at the bottom of the body domain. The density is absent at the bottom of *Msm*30S where h17 packs against h10 in *E. coli* 30S subunit (Figure 2). The sequence alignment of *M. smegmatis* and *M. tuberculosis* 16S rRNAs does not show major insertion/deletion. Hence, in the absence of *M. smegmatis* rRNA secondary structures, we inspected the available 16S rRNA secondary structure of *M. tuberculosis* (Figure 2A; database: www.rna.ccbb.utexas.edu). The 16S rRNA secondary structure indeed shows that h10 and h17 are substantially shorter in mycobacteria relative to the same of *E. coli*.

Additional density clusters are distributed over the head, platform and body regions of the solvent side of *Msm*30S (Figure 2B). Data mining and bioinformatics analysis of the sequences revealed that some of the mycobacterium ribosomal proteins are significantly bigger as compared to their *E. coli* homologs (Table 1). Interestingly, these proteins are anchored on the solvent-exposed surfaces of the subunits. The positions of the major extra density clusters located in the head and upper part of the body of the *Msm*30S are in good agreement with the locations of S3, S5, and S9 proteins, allowing the tentative identification of the additional segments of these proteins (Figure 2B).

In contrast to h10, h9 is longer in mycobacteria than its *E. coli* counterpart. Proteins S16 and S17 adjacent to h9 are also bigger than their *E. coli* homologs (Table 1). An additional bulky density at the lower body region in *Msm*30S (marked with asterisk in Figure 2B) is visible that can be accounted for by the extra segments of h9, as well as S16 and S17 proteins.

Differences are seen in the shapes and sizes of the spur and the beak regions. The corresponding rRNA elements, h6 and h33a respectively, are truncated in mycobacterial 16S rRNA as compared to those of the *E. coli*, thereby resulting the overall curvatures of the spur and the beak different in *Msm*30S (Figure 1,2).

These rRNA regions, where marked differences are seen, have been identified as variable regions in 16S rRNA [16], and differences in size are expected to be observed. Our study reveals how these differences in secondary structures reflect in the 3D structure of the *Msm*70S.

### Additional structural features in the 50S subunit

The 50S subunit of *Msm*70S (*Msm*50S) also contains several extra densities located at its periphery and solvent-exposed side (Figure 3B). The sequence alignment of different mycobacterial 23S rRNAs also suggests that there is no significant difference exists in the 23S rRNA secondary structures of different mycobacterial species. Several extra helices are present in the 23S rRNA of mycobacteria (Figure 3A; database: www.rna.ccbb.utexus.edu). Interestingly, the salient features are concentrated in the 5′ half of the 23S rRNA (domains I, II and III), that occupies the solvent-exposed surface of the 50S subunits [17]. Most of the additional density of the *Msm*50S can largely be attributed to the extra helices present in the 23S rRNA.

The most conspicuous structural feature in *Msm*50S is a slender rod-like additional density (which we term the ‘steeple’) that emerges from the bottom of a lateral side of *Msm*50S and runs up at the L1 protuberance (Figure 3B). The steeple measures ∼160 Å in length with the upper end forming a pointed structure.

**Figure 4:**
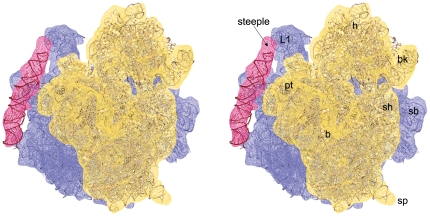
Location of the steeple (H54a). Stereo representation of the density map of steeple is shown in wire mesh (deep pink) with 50S (blue) and 30S subunit (yellow) to show its orientation relative to the *Msm*70S subunits. Atomic coordinates of the *E. coli* 30S (pdb code: 2I2U) and 50S (pdb code: 2I2V) subunits docked into the corresponding density maps are also shown . A 96 nucleotide long rRNA helix has also been fitted into the density of steeple. Landmarks for the 30S subunit: bk, beak; h, head; pt, platform; sh, shoulder; sp, spur; b, body. Landmarks for the 50S subunit: L1, L1 protein; sb, L7/L12 stalk base.

**Figure 5:**
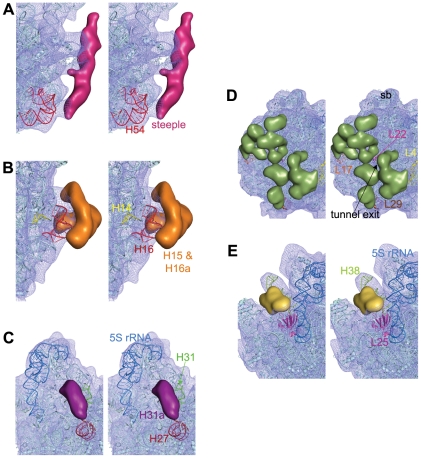
Identification of the extra rRNA helices and additional segments of r-proteins in the *Msm*50S. Close-up views of the (A) H54 region showing that the steeple (deep pink) emerges from H54, (B) H14 and H16 region displaying that the bifurcated density (orange) is related to these helices, and (C) H31 region identifying the density corresponding to the extra helix (purple), H31a, in the 23S rRNA, are shown in stereo. Density clusters attributable to extra domains of *M. smegmatis* ribosomal large subunit proteins L4/L17/L22/L29 (D), and L25 (E) are also shown in stereo. *T. thermophilus* L25 (from pdb code 2J01, chain Z, 3–179 amino acids) protein structure is shown here (magenta) to identify the C-terminal domain. Part of the C-terminal extra domain overlaps with the density cluster attributed to L25 in *Msm*70S.

Strikingly, this region in the yeast 80S ribosome is very similar in appearance. The main helix of expansion segment 27 (ES27) of 25S rRNA (domain IV, [18]), one of the rRNA insertions characteristic for 80S ribosomes, makes similar helical feature (∼150 Å long) (termed ‘yeast-spine’ [19]) in the back of *S. cerevisiae* 60S subunit close to L1 arm. Superimposition of yeast 60S subunit [18] and the *Msm*50S revealed that the yeast spine originates at a lower region of the yeast 60S subunit compared to the steeple of the *Msm*50S (see Figure S2A). The base of the steeple, in contrast, juxtaposes on the ES26 (designated as insertion at H54) of the yeast 25S rRNA (domain III). As a result, the upper end of the steeple reaches much closer to the L1 stalk and the shoulder of the 30S subunit as compared to the yeast spine (Figure 4). Indeed, a long extra helix (marked as H54a in Figure 3A) following helix H54 exists in the secondary structure of mycobacterial 23S rRNA. Helix H54a contains ∼110 nucleotides. Docking of a 96 nucleotide long RNA helix (taken from pdb code: 2XKV) inside the steeple density (Figure 4) matched the length reasonably well (cross-correlation coefficient 0.70). Docking of the rRNA crystal structure of *E.coli* 50S subunit into the *Msm*50S map allowed us to unambiguously identify the steeple as the extra helix, H54a (Figure 5A). Mining of database revealed that this extra helix exists in some other members of Actinobacteria like *Frankia*, *Micrococcus*, *Rhodococcus* and *Streptomyces*.

**Figure 6:**
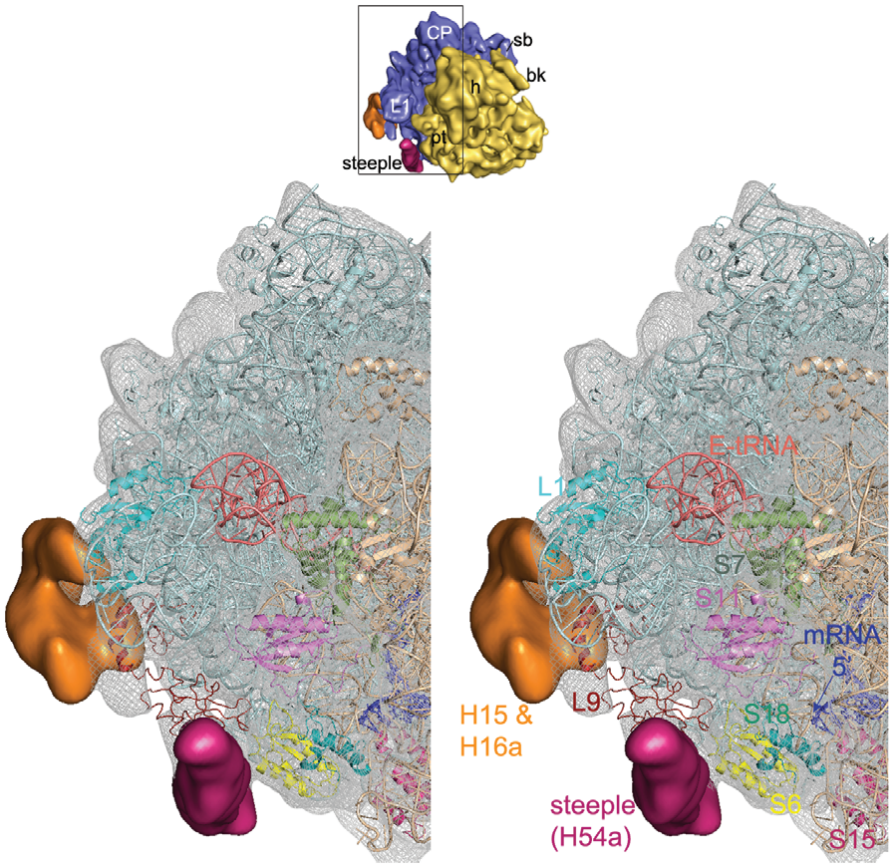
Neighbourhood of the steeple structure. Close-up view of the steeple (deep pink) is shown with its neighbouring ribosomal proteins and rRNA structures in stereo. The *Msm*70S density map is represented in grey wire mesh with the coordinates of the 30S (Wheat colour) and the 50S (light cyan) subunits docked into the map. Coordinate of mRNA (blue stick model) is taken from a crystal structure [46] of the *T. thermophilus* 70S ribosome (pdb code: 2HGR, chain 1) and aligned to the atomic structures used here. A thumbnail view of the Msm70S is shown on top to orient the reader.

Besides the steeple structure, additional density clusters are also present in the solvent side of the large subunit of *Msm*70S (Figure 3B). As indicated in the secondary structure (Figure 3A), *Mycobacterium* 23S rRNA possesses three more extra helices as insertions at H14, H16, and H31 (designated as H15 (as marked in the *H. marismortui* 50S subunit structure [17]), H16a, and H31a in Figure 3A), in addition to H54a. The H15 and H16a are placed at the solvent side of the *Msm*50S, appearing to branch into two irregular helices (Figure 5B). The larger branch runs toward the base of the L1 stalk and appears to interact with the N-terminal of the L9 protein (Figure 6). Density attributable to H31a is also visible (Figure 5C) at the solvent side of the *Msm*50S below the central protuberance (CP).

The bottom surface of the *Msm*50S, which includes the exit of polypeptide tunnel, is studded with extra density (Figure 3B). Proteins L17, L22 and L29 that surround the tunnel exit and L4 are significantly bigger in *M. smegmatis* than their *E. coli* counterparts (Table 1). Majority of the extra density in this region may be accounted for by the additional domains of these proteins (Figure 5D). We suggest that the extra domains of the proteins around the tunnel exit may play a role in coordinating access of nonribosomal factors, such as ribosome-associated chaperones, signal recognition particle (SRP), or the translocon to the tunnel exit site and thereby to the emerging nascent chain. In addition, they may interact directly with the nascent chain or with nascent chain-interacting proteins.

Another interesting feature is visible at the groove formed by the CP and the base of L7/L12 stalk. H38 in this region looks expanded due to the presence of a large piece of density adjacent to the helix (Figure 5E). The protein in the immediate neighbourhood of this helix, L25, in *M. smegmatis* is much longer (121 amino acids more at the C-terminal) than its *E. coli* homolog (94 amino acids). Thus the extra density must be attributed to the extra part of the protein L25. L25 in *T. thermophilus* is also 206 amino acids long, and the position of its C-terminal domain in the crystal structure [20] is in agreement with our localization of the extra domain of L25 in the *Msm*50S. The tail of the protein L25, adjacent to H38, appears to be of mechanistic interest because of the prominent role of H38 in the formation of a bridge (b1a) with the 30S subunit and in forming a contact with the elbow of the A site-bound tRNA (A site finger) [21]. It appears that L25 participates in the regulation of H38 movements [22] in *Msm*70S.

Major parts of the additional density present in *Msm*30S and *Msm*50S (Figure 2, 3) are accounted for by the positions of the additional rRNA helices, as well as additional domains in the homologous proteins. Nevertheless, extra density regions of unknown identity also exist in both the subunits of *Msm*70S. It should be noted at this point that studies in the seventies identified an antigen (designated as beta antigen), common to all mycobacteria, which is ribosomal. It was suggested that the beta antigen is a constituent of the ribosome [3], [4], [23]. Therefore, it is plausible that part of the unaccounted additional density in the *Msm*70S is attributable to the beta antigen. However, in the absence of additional information, localization of this antigen is not possible.

### Implications for the translation process

The structural components visible as additional mass in the 3D structure of *Msm*70S appear to be engaged in additional tertiary and quaternary interactions. The most intriguing of the extra elements is the steeple. In the current map the steeple is tilted toward the platform of the 30S subunit and the pointed tip of the steeple structure is located in the immediate vicinity of the mRNA exit channel. The distances between the tip of the steeple and the mRNA exit, L1 stalk, and E-site tRNA are ≈50 Å, 35 Å, and 65 Å respectively. The middle part of the steeple interacts with the C-terminal of L9, and forms a unique bridge with the protein S6 of the 30S subunit (Figure 6).

In the *Msm*70S map the steeple apparently blocks the exit path of the E-site tRNA and likely act as a gate for the E-site tRNA. The freestanding conformation of the steeple would allow a certain freedom of movement. Thus, it is possible that this helical structure alternates between a ‘closed’ conformation, as seen in the current map, and an ‘open’ conformation, where the exit path of the E-site tRNA is free. Previous cryo-EM studies on *Eco*70S have suggested that the L1 stalk actively participates in the translocation process [24]. In mycobacteria, along with the L1 stalk, the steeple appears to participate in the exit process of the E-site tRNA. Therefore, a legitimate guess would be that the dynamics of this helix may facilitate efficient exit of the E site tRNA. Ratchet-like rotation of the small vs. large subunit [25] has been postulated to be an integrated part of the tRNA translocation mechanism [24], [26], [27]. We have aligned a cryo-EM map of the EF-G-bound *E. coli* 70S ribosome (EMD-1363) in ratcheted state with the Msm70S map (Figure S2B). The small subunit movement apparently disrupts the bridge between the steeple and the S6 protein. Thus, the 30S subunit conformational change due to ratchet motion would most likely facilitate the movement of the steeple.

The close proximity of the steeple to the mRNA exit channel (Figure 4, 6), observed in the current map, led us to propose that the steeple may be involved in modulating the initiation process in mycobacteria. In this context, it is noteworthy that the platform region, which is in the immediate neighborhood of the tip of the steeple, is a crucial center for the binding and adaptation of the mRNA during the initiation [28], [29]. Although the localization of the domains (N- and C-terminals) of IF3 is still controversial, the structural studies [30], [31] have identified the platform of 30S subunit as the IF3 binding region. It is tempting to propose that the steeple structure may play a role in modulating interactions of cis- and trans-acting regulators involved in translation initiation [32]. Other possibility that the steeple may interact directly with the progressing mRNA 5′ end can not be ruled out. Interestingly, the S1 protein is much smaller in mycobacteria (479 aa) as compared to that of the *E. coli* (557 aa). It appears from sequence alignment that a whole C-terminal domain is missing in mycobacterial S1. As evident from the biochemical studies, S1 protein has a crucial role in stabilizing mRNAs [33]–[35]. It is possible that in the absence of a C-terminal domain in S1, the steeple plays a similar role in mycobacterial ribosome.

The bacterial ribosome structures elucidated so far are very similar. In contrast, the archaeal [17], eukaryotic (cytoplasmic) [18], or organellar ribosomes [36] show extra features that are proposed to have crucial functional roles [36], [37], [38]. The mycobacterial ribosome described in this study is the first structure showing structural diversity in bacterial ribosomes. Thus, it is conceivable that the extra features, particularly the steeple (considering the location of this unique helix), possess an active role in different steps of the translation process in mycobacteria.

Overall, the mechanism of translation may be well conserved between *E. coli* and *M. smegmatis* as concluded in a recent biochemical study [39], our results indicate that the intricate details of the mechanisms of various steps likely differ in mycobacterium due to the involvement of its unusual structural features.

## Materials and Methods

### Purification of the M smegmatis 70S Ribosome

*M. smegmatis* cells were grown in 2XYT medium supplemented with 0.5% glycerol, 0.2% Tween-80 and 1% glucose at 37°C until OD_600_ reach to 0.8. 70S ribosomes are purified following the method of *E. coli* 70S purification [40] with modifications. Harvested cells (checked by Ziehl-Neelsen staining method to check presence of any contaminating bacteria) were washed with BufferA (20 mM Tris-HCl, pH 7.5, 10 mM Magnesium acetate, 100 mM Ammonium chloride, 5 mM 2-Mercaptoethanol) and lysed at 30 K psi using a Cell Disruption System (Constant Cell Disruption Systems, United Kingdom) in the same buffer where DNase I (2 mg/ml) was added. Cell lysate was clarified by centrifuging in a SIGMA 12158-H rotor at 15000 *g* for several times till no pellet was seen. Supernatant was subjected to ultracentrifugation at 4°C at 154000 *g* in a Sorvall AH-629 rotor for 2 hrs and pellet resuspended in TMA10 buffer (Tris-HCl, pH 7.5, 10 mM Magnesium acetate, 30 mM Ammonium chloride, 5 mM 2-Mercaptoethanol). The suspension was homogenized for 1 hr in presence of 1 M ammonium chloride after which the ribosomal preparation was centrifuged for several times at 4°C at 15000 *g* in a SIGMA 12158-H rotor till no pellet was seen. Supernatant solution was centrifuged in a Sorvall AH-629 rotor at 4°C at 154000 *g* for 2 hr. Supernatant discarded, pellet collected, resuspended in TMA10 buffer. Sample was loaded on top of a 5%–20% linear sucrose gradient in TMA10 and centrifuged in a Sorvall AH-629 rotor at 154000 *g* for 90 min at 4°C. Gradient collected from bottom to top. Relevant fractions were pooled together. To this 7.5 M ammonium acetate and chilled ethanol were added and kept at −80°C overnight and centrifuged at 4°C at 12000 rpm in a SIGMA 12155-H rotor. Pellet of purified 70S particle was resuspended in TMA10 and concentration and purity of ribosome samples were measured and stored at −80°C.

### Cryo-electron microscopy (cryo-EM) and Image Processing

Buffer solutions containing *M smegmatis* 70S ribosome with final concentration of 32 nM were applied to grids and cryo-EM grids were prepared according to standard procedures [41]. Data were collected using 4 K×4 K CCD camera with a physical pixel-size of 15 um (corresponding to a pixel size of 1.69 Å on the object scale) on a Philips FEI (Eindhoven, The Netherlands) Tecnai F20 field emission gun electron microscope equipped with low-dose kit and an Oxford cryo-transfer holder at a total magnification of 89,000×.

From a total number of 268 micrographs, after evaluation of drift, astigmatism, and the presence of Thon rings in the power spectrum of each micrograph, 215 good micrographs were selected and divided into 50 defocus groups (ranging from ∼1.5–3.7 µm). Ribosomal particles were selected from these micrographs through three steps: preliminary automated selection, manual verification, and selection based on the size of the cross-correlation coefficient with a template.

Since, as a bacterial ribosome, the *Mycobacterium* ribosome is expected to display overall morphological similarity to the *E. coli* ribosome, we initially used a 6.7 Å resolution map of *E. coli* 70S ribosome (EMD-5036 [42]) as the reference to obtain a 13.6 Å map of the *M smegmatis* ribosome using 48,708 images. The newly reconstructed volume of *M smegmatis* 70S ribosome was then used as reference volume applied to the entire data set for the final reconstruction. The final resolution of the CTF-corrected volume, estimated by the Fourier Shell Correlation (FSC) criterion with a cutoff value of 0.5, was 12 Å (Figure S1). The falloff of the Fourier amplitudes toward higher spatial frequencies was corrected as described [43], using the x-ray solution scattering intensity distribution of the *E. coli* 70S ribosome.

All of the steps of image processing were performed by using the SPIDER package [44].

### Molecular modelling and visualization

Crystal structures [15] of the small (PDB code: 2I2U) and the large subunit (PDB code: 2I2V) of *E. coli* 70S ribosome were docked (as rigid body) manually into the cryo-EM density map of *M. smegmatis* 70S ribosome using PyMol (DeLano Scientific).

SPIDER was used for the computational separation of the cryo-EM reconstructions into densities corresponding to individual ribosomal subunits or extra densities in *M smegmatis* 70S ribosome. Difference mapping computed by subtracting the map of each subunit of the *E.coli* 70S ribosome from the same of the *M. smegmatis* 70S ribosome produced extra densities related to the subunits of *M. smegmatis* 70S ribosome. Isolation of each of the major density clusters was performed by selecting the mass of interest from the remaining masses using a clustering procedure.

Comparison of the ribosomal protein sizes of *M. smegamatis* to that of the *E. coli* was made in protein blast server (http://blast.ncbi.nlm.nih.gov/) using Blastp algorithm. Visualization and preparation of illustrations were done using Pymol and Chimera [45].

### Accession number

The cryo-EM map of the 70S ribosome from *M. smegmatis* has been deposited in the 3D-EM Database (RCSB EMDB site (http://www.emdatabank.org)) with accession code EMD-5307.

## Supporting Information

Figure S1 **Resolution curve.** Fourier shell correlation (FSC) curve for the cryo-EM map of the *Msm*70S. FSC = 0.5 indicates 12 Å resolution.
(TIF)

Figure S2 **Position of the Steeple.** (A) The density map (40S subunit sand colour and 60S subunit cyan) of the yeast 80S ribosome (EMD-1076) is superposed on the *Msm*70S (30S subunit yellow and 50S subunit blue). The difference in the locations of the steeple (deep pink) and the yeast-spine (marked) is clearly visible. Landmarks: CP, central protuberance; L1, L1 protein; h, head; pt, platform; bk, beak; sh, shoulder; b, body. (B) The density map (EMD-1363) of the *E. coli* 70S ribosome in ratcheted state (30S subunit in sand colour and 50S subunit in cyan) is superimposed on the *Msm*70S (30S subunit yellow and 50S subunit blue) map. The bridge formed by the steeple and the protein S6 in *Msm*70S (marked with white arrow) apparently gets disconnected due to the ratchet motion of the 30S subunit.
(TIF)
