# Supplementary figures and images for: Structural Diversity in Bacterial Ribosomes: Mycobacterial 70S Ribosome Structure Reveals Novel Features
Source: PLoS One. 2012 Feb 24;7(2):e31742. doi: 10.1371/journal.pone.0031742 (PMC3286452; doi:10.1371/journal.pone.0031742)

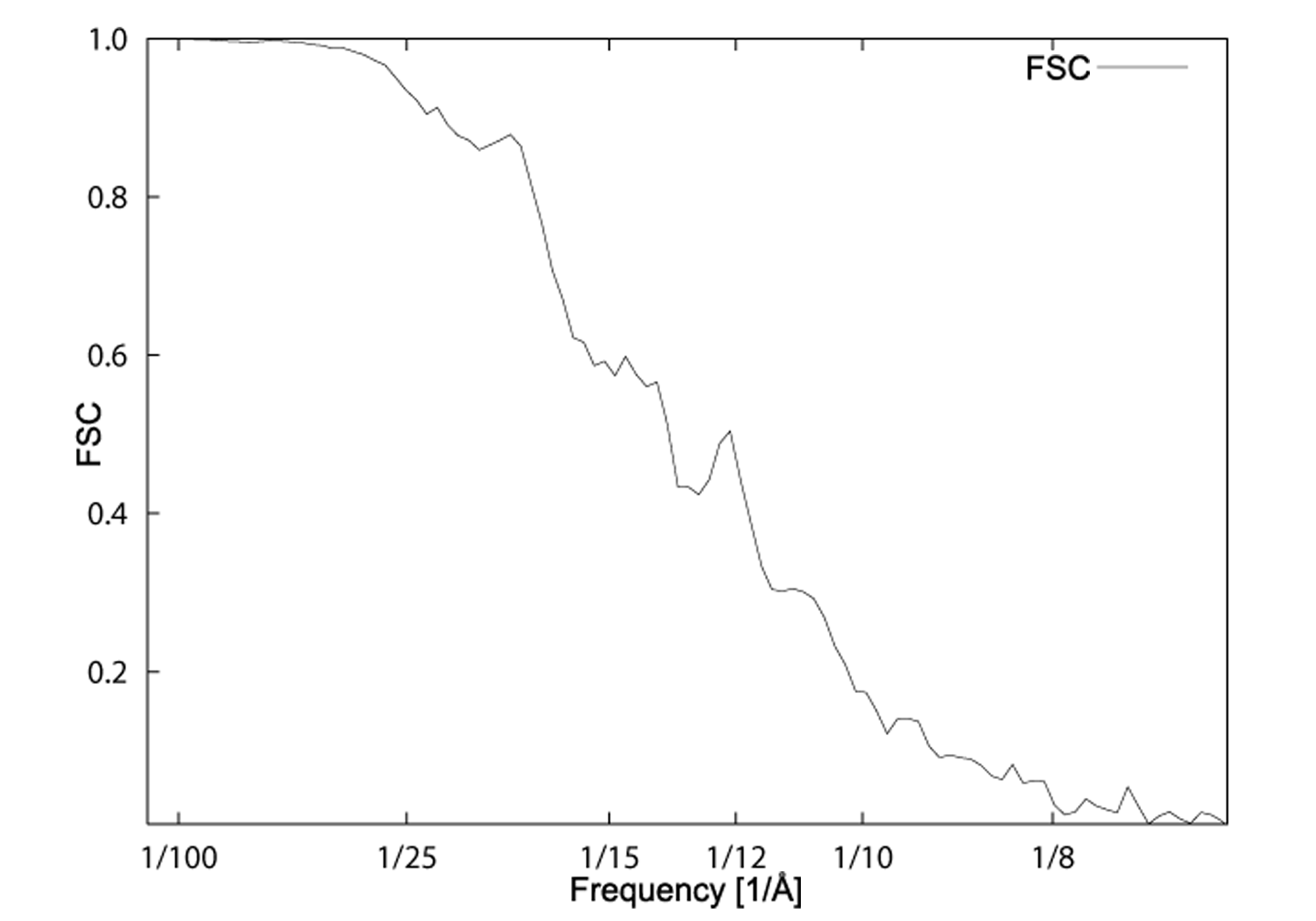

Supplement: Figure S1 — Resolution curve. Fourier shell correlation (FSC) curve for the cryo-EM map of the Msm70S. FSC = 0.5 indicates 12 Å resolution. (TIF) [file pone.0031742.s001.tif]

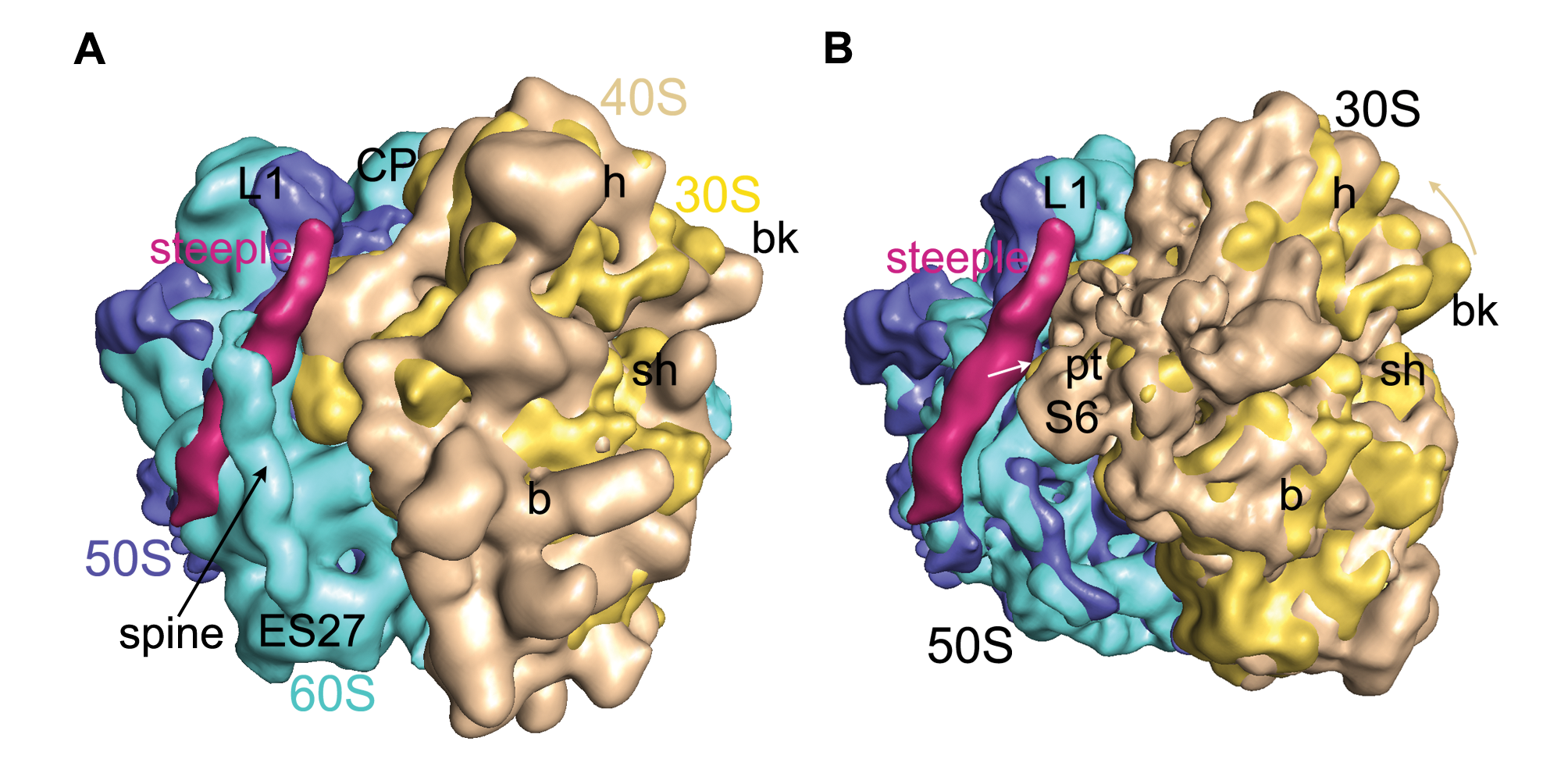

Supplement: Figure S2 — Position of the Steeple. (A) The density map (40S subunit sand colour and 60S subunit cyan) of the yeast 80S ribosome (EMD-1076) is superposed on the Msm70S (30S subunit yellow and 50S subunit blue). The difference in the locations of the steeple (deep pink) and the yeast-spine (marked) is clearly visible. Landmarks: CP, central protuberance; L1, L1 protein; h, head; pt, platform; bk, beak; sh, shoulder; b, body. (B) The density map (EMD-1363) of the E. coli 70S ribosome in ratcheted state (30S subunit in sand colour and 50S subunit in cyan) is superimposed on the Msm70S (30S subunit yellow and 50S subunit blue) map. The bridge formed by the steeple and the protein S6 in Msm70S (marked with white arrow) apparently gets disconnected due to the ratchet motion of the 30S subunit. (TIF) [file pone.0031742.s002.tif]
